# Arthroscopic Posterior Cruciate Ligament Reconstruction Using a Pedicled Semitendinosus Autograft Through a Trans‐Septal Approach: A Technical Note

**DOI:** 10.1002/atn2.70194

**Published:** 2026-07-14

**Authors:** Ali Alayane, Vincent Marot, Dany Mouarbes, Léon Ortal, Ariane Le Cunf, Regis Pailhe, Etienne Cavaignac

**Affiliations:** ^1^ Clinique Universitaire du Sport, Centre Hospitalier Universitaire de Toulouse (CHU) Toulouse France; ^2^ Centre Hospitalier de Perpignan (CHP) Perpignan France; ^3^ Cabinet Arthropole Bayonne France

## Abstract

Isolated posterior cruciate ligament (PCL) ruptures are relatively uncommon among knee ligament injuries. Operative management is recommended for patients with grade III posterior knee joint laxity. The complex anatomy of the PCL, particularly its course and insertion on the femur and the tibia, makes its reconstruction technically challenging. In this article, we describe a surgical technique for anatomical reconstruction of the PCL using a pedicled semitendinosus tendon autograft. The procedure is performed through trans‐septal approach with preservation of the native PCL remnants. Our technique aims to achieve a biological PCL reconstruction, enhancing graft vascularization and promoting optimal healing.

## Graphical abstract

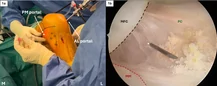

**VIDEO 1:** This instructional video demonstrates an arthroscopic technique for the posterior cruciate ligament (PCL) reconstruction using a pedicled semitendinosus tendon autograft. With the patient is the supine position and the knee joint flexed to 90°, the first stage consists of creating posteromedial and posterolateral portals using the trans‐septal approach. The second stage involves preparing the PCL tibial tunnel under direct arthroscopic visualization through the posteromedial portal with a shaver introduced through the posterolateral portal. The femoral tunnel in then created using an accessory anterolateral portal under direct arthroscopic visualization through the anterolateral portal. The semitendinosus tendon is harvested in the standard manner and left attached to its tibial insertion. After accurate measurement of the tibial and femoral tunnel lengths, a tripled pedicled semitendinosus graft is prepared. Keys steps include the trans‐septal approach, anatomical tibial tunnel footprint preparation, and preservation of the PCL remnants. The goal of this procedure is to achieve an anatomical and biological PCL reconstruction using a pedicled semitendinosus graft with triple tibial fixation, while preserving graft vascularity to promote optimal integration and healing. Video content can be viewed at https://doi.org/10.1002/atn2.70194.

The posterior cruciate ligament (PCL) has a crucial role in controlling posterior tibial translation and external tibial rotation.^1^ PCL ruptures are uncommon, representing approximately 3% of all knee injuries and often occur in the context of multiligament knee injuries.^2^, ^3^ This injury commonly results from a posteriorly directed force applied to the proximal tibia with the knee flexed, such as during motor vehicle accident or a fall onto a flexed knee with the foot in plantar flexion.^4^ Although most isolated PCL injuries can be treated nonoperatively, surgical reconstruction is typically reserved for patients with significant posterior instability following failure of conservative treatment, with the goal of restoring tibiofemoral kinematics and preventing secondary osteoarthritis of the medial or patellofemoral knee compartments.^4^, ^5^ PCL reconstruction remains a technically demanding procedure necessitating advanced arthroscopic skills. The challenges related to this surgery arise from multiple factors including graft selection, adequate visualization of the posterior knee compartments, preservation of native PCL remnants, prevention of popliteal neurovascular structures injury, reduction of the Sharpe angulation between the tibial and femoral tunnels commonly referred to as the “killer turn” during graft passage, and achievement of secure graft fixation.^6^, ^7^

Many techniques have been described for PCL reconstruction aiming to restore sagittal knee joint stability and preserve the PCL remnants.^6^, ^8^, ^9^ The authors describe a biological technique for PCL reconstruction using a pediculed semitendinosus (ST) tendon autograft. The objective of our technique is to preserve the native attachment of the ST tendon, thereby maintaining graft vascularity and promoting biological healing as previously shown in pedicled ACL reconstruction.^10^ Moreover, the trans‐septal approach facilitated clear visualization and preservation of the PCL remnants, which further supports optimal graft integration and healing.^11^ Pearls and pitfalls are described in Table 1. Advantages and disadvantages are presented in Table 2.

**TABLE 1:** Surgical Steps, Pearls, and Pitfalls of Arthroscopic Posterior Cruciate Ligament Reconstruction Using a Pedicled Semitendinosus Graft With Trans‐Septal Visualization

Surgical Steps	Pearls	Pitfalls
Trans‐septal approach	Precise creation of the posterior portals using needle guidance under direct skin transillumination	Gentle debridement of the septum using the shaver, while keeping the cutting edge always directed toward the bone to prevent posterior neurovascular injury
	Careful separation of the posterior capsule from the PCL footprint, aiming to maintain PCL remnants to enhance biological graft healing	
PCL tibial and femoral tunnels creation	Arthroscopic visualization of the PCL remnants achieved by inserting the arthroscope through the PM portal to ensure anatomical tibial tunnel positioning	The guidewire should be maintained by a holder during tibial tunnel over drilling to prevent displacement and penetration into the posterior capsule, thereby reducing the risk of neurovascular injury
Pedicled PCL graft Preparation	Accurate determination of the tunnel lengths under direct arthroscopic visualization is fundamental to achieve proper graft tension and optimal tunnels graft filling	
PCL graft passage and femoral fixation	The Tightrope is pulled through the PCL femoral tunnel under direct arthroscopic visualization via the AM portal to ensure adequate passage and tightening on the medial femoral epicondyle before pulling the PCL graft into the femoral tunnel	Graft visualization through the PM portal is recommended during graft passage, using a hook introduced through the PL portal to guide the graft and avoid the “killer turn”
PCL graft tibial fixation	Maintaining the K‐wire during tibial screws fixation for tibial fixation is essential to prevent iatrogenic injury of the posterior neurovascular structures	PCL graft fixation should be performed with the knee flexed at 90° in a reduced position to minimize the risk of residual posterior knee laxity
	Triple PLC tibial fixation is achieved using 2 interference screws and the native ST tendon tibial attachment. The first screw reaches the posterior tibial cortex, while the second screw is flush to the anterior tibial cortex ensuring accurate graft tensioning and fixation	

AM, anteromedial; PCL, posterior cruciate ligament; PL, posterolateral portal; PLC, posterolateral corner; PM, posteromedial portal.

**TABLE 2:** Advantages and Disadvantages of Posterior Cruciate Ligament Reconstruction Using a Pedicled Semitendinosus Tendon With a Trans‐Septal Approach

Advantages	Disadvantages
Arthroscopic anatomic PCL reconstruction with accurate visualization of the PCL tibial tunnel footprint, allowing precise and anatomic tunnel placement	The trans‐septal approach requires a considerable learning curve
Elimination of the need for intraoperative fluoroscopic guidance during tibial tunnel preparation, reducing radiation exposure and simplifying patient positioning and draping	This technique is unfeasible if the hamstring tendons are absent or insufficient in length or diameter to create a tripled graft
Direct visualization of the PCL graft through the PM portal, enabling accurate graft passage and minimizing the risk of the “killer turn” during graft passage	Because the pedicled graft is prepared according to the measured tibial and femoral tunnel lengths, this preparation occurs only after tunnel's creation. Therefore, tunnel sizing cannot be based on the graft diameter. In practice the final graft diameter is generally around 8 mm in females and 9 mm in males
Triple PCL graft fixation using 2 bioabsorbable screws, While preserving the semitendinosus tendon tibial attachment, to ensure secure and rigid tibial fixation until graft integration and healing occur	Risk of popliteal neurovascular injury during tibial tunnel creation
Optimized biological graft healing is obtained by maintaining the semitendinosus graft attached to its native tibial insertion and preserving the PCL remnants and as well as the meniscofemoral ligament	

PCL, posterior cruciate ligament; PM, posteromedial portal.

## SURGICAL TECHNIQUE

### Patient Setup

Under general anesthesia, the patient is placed in the supine position with a tourniquet secured around the operative thigh. The knee joint is assessed to ensure full range of motion and stability while resting on a foot roll at 90° of flexion. Posterior drawer test is performed preoperatively to confirm posterior knee instability (Video 1).

### Trans‐Septal Approach and Posterior Portals Creation

A ST tendon autograft is harvested in the standard manner and left attached to its tibial insertion. It is then protected with a gauze soaked in vancomycin antibiotics as a precautionary measure to minimize the risk of infection.

A diagnostic arthroscopy is performed through standard anterolateral, and anteromedial (AM) portals using a 30° arthroscope to assess and identify any associated intra‐articular pathology. Using the trans‐notch approach, the posteromedial (PM) portal is created in the standard manner (Figure 1). The lateral aspect of the septum is then visualized, and septal debridement is performed using a shaver introduced through the PM portal to create the trans‐septal opening (Figure 2). Subsequently, the arthroscope is inserted through the PM portal and advanced via the septal window to establish the posterolateral portal under direct visualization, assisted by transillumination of the skin (Figure 3).

**FIGURE 1:**
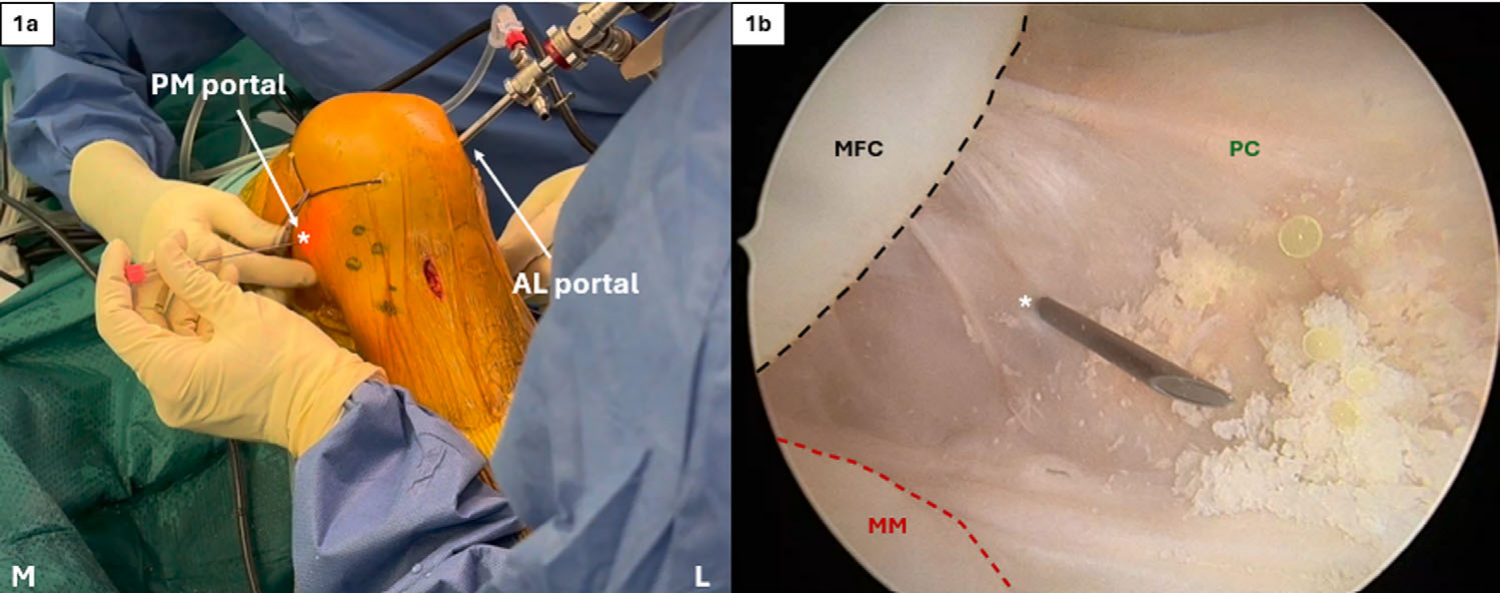
Intraoperative photograph of the left knee in 90° of flexion showing the creation of the PM (*) portal using an 18‐gauge needle, with the arthroscope inserted in the PM knee compartment via the trans‐notch approach (a). Arthroscopic visualization through the AL portal of the PM compartment showing the needle entry point for PM portal creation (b). (AL, anterolateral portal; L, lateral side; M, medial side; MFC, medial femoral condyle; MM, medial meniscus; PC, posterior capsule; PM, posteromedial portal.)

**FIGURE 2:**
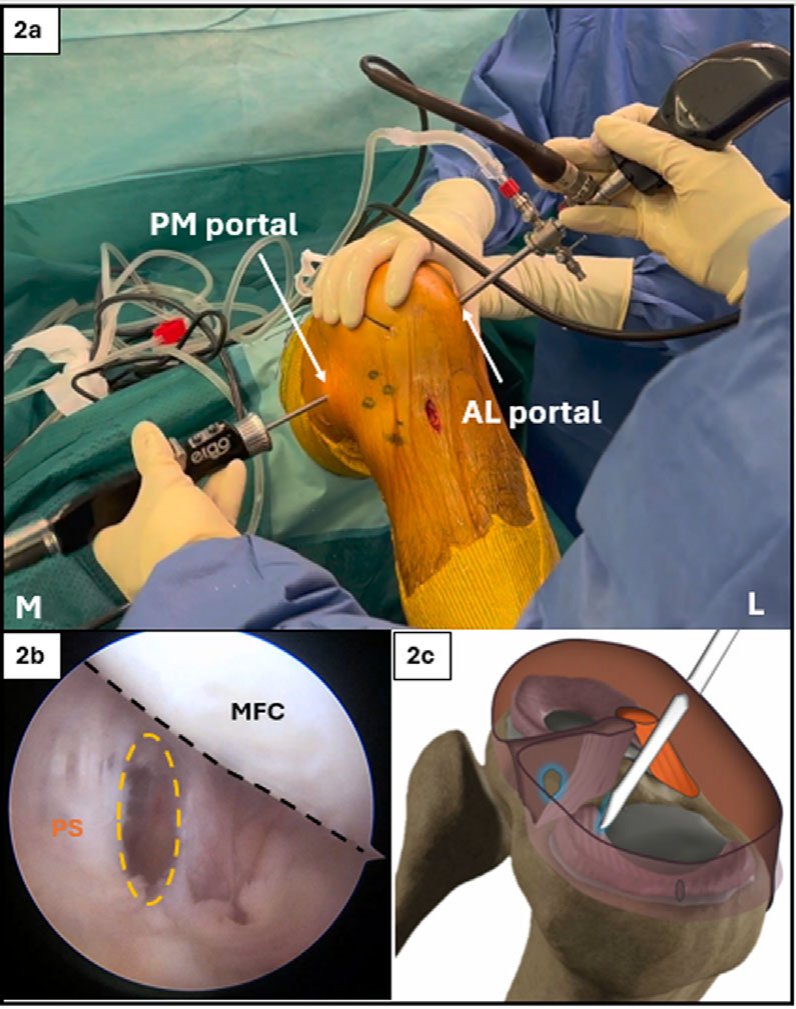
Intraoperative photograph of the left knee with the patient showing the creation of the trans‐septal opening using a shaver introduced through the PM portal under arthroscopic visualization through the trans‐septal approach. The cutting surface of the shaver is consistently oriented toward the bone to prevent potential popliteal vascular injury during posterior septal opening creation (a). Arthroscopic view of the left knee in 90° of flexion with the arthroscope inserted through the PM portal showing the opening of the posterior septum to create the trans‐septal approach (b). Schematic presentation of the posterior septal opening creation (c). (AL, anterolateral portal; L, lateral side; M, medial side; MFC, medial femoral condyle; PM, posteromedial portal; PS, posterior septum.)

**FIGURE 3:**
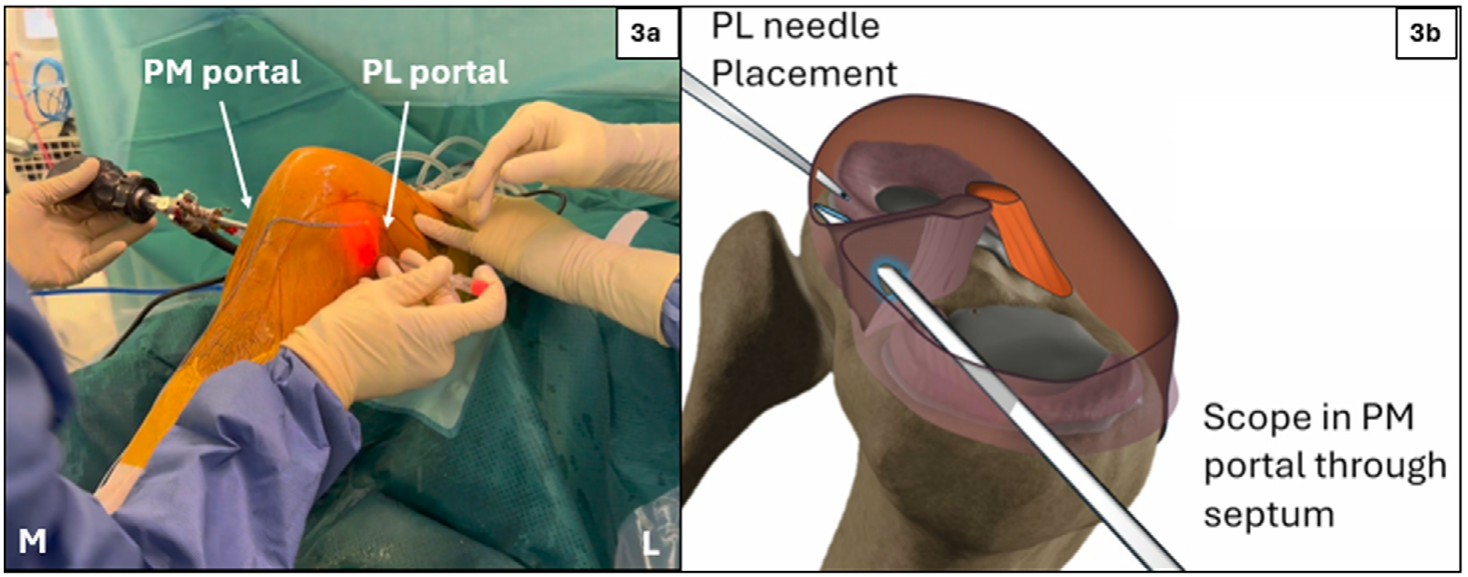
Intraoperative photograph of the left knee in 90° of flexion showing creation of the PL under direct skin transillumination, with the arthroscope introduced through the PM portal and the septal opening reaching the posterolateral compartment (a). Schematic presentation of the posterolateral approach creation (b). (L, lateral side; M, medial side; PL, posterolateral portal; PM, posteromedial portal.)

Through the posterolateral portal, a shaver is used to release the septum from the posterior capsule, allowing visualization of the PCL tibial remnants, which serve as a footprint for tibial tunnel creation (Figure 4). The cutting surface of the shaver is consistently oriented toward the bone to avoid potential popliteal vascular injury (Video 1).

**FIGURE 4:**
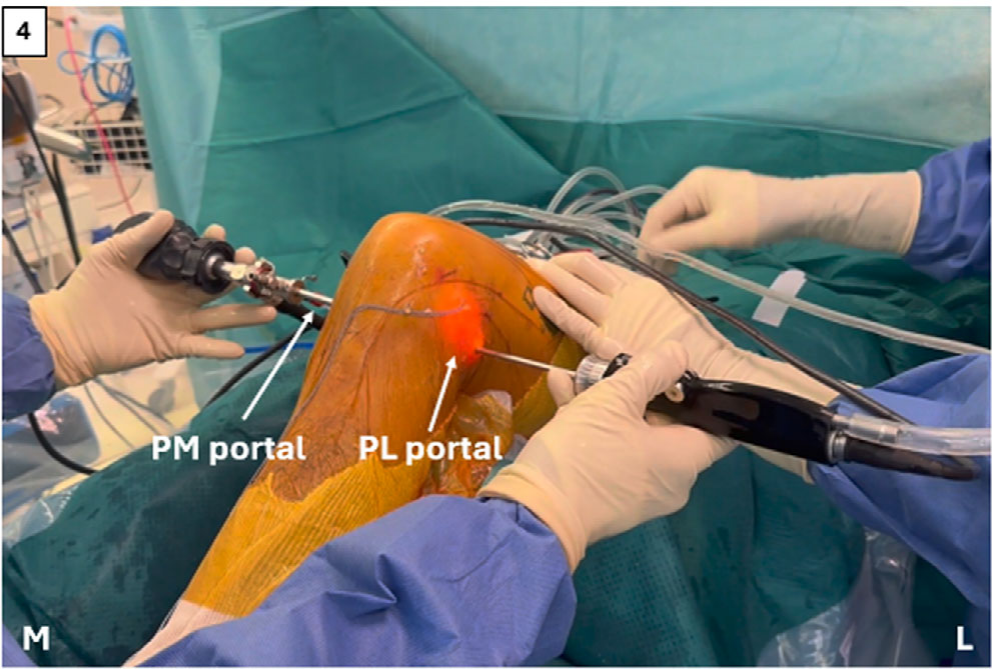
Intraoperative photograph of the left knee in 90° flexion, showing the preparation of the PCL footprint with the arthroscope introduced through the PM portal and the shaver through the PL portal. The cutting surface of the shaver is consistently oriented toward the bone to avoid potential popliteal vascular injury. (L, lateral side; M, medial side; PCL, posterior cruciate ligament; PL, posterolateral portal; PM, posteromedial portal.)

### PCL Femoral and Tibial Tunnels Creation

Through an accessory anterolateral portal, a guide wire is inserted into the femoral footprint of the PCL, approximately 10 mm posterior to the medial femoral condyle articular cartilage and directed proximally and slightly posteriorly to the lateral femoral epicondyle (Figure 5). It is then over‐drilled from inside to outside using a 9 mm drill‐bit to create a 20 mm length femoral tunnel. A loop suture is subsequently placed with the tunnel for later graft passage.

**FIGURE 5:**
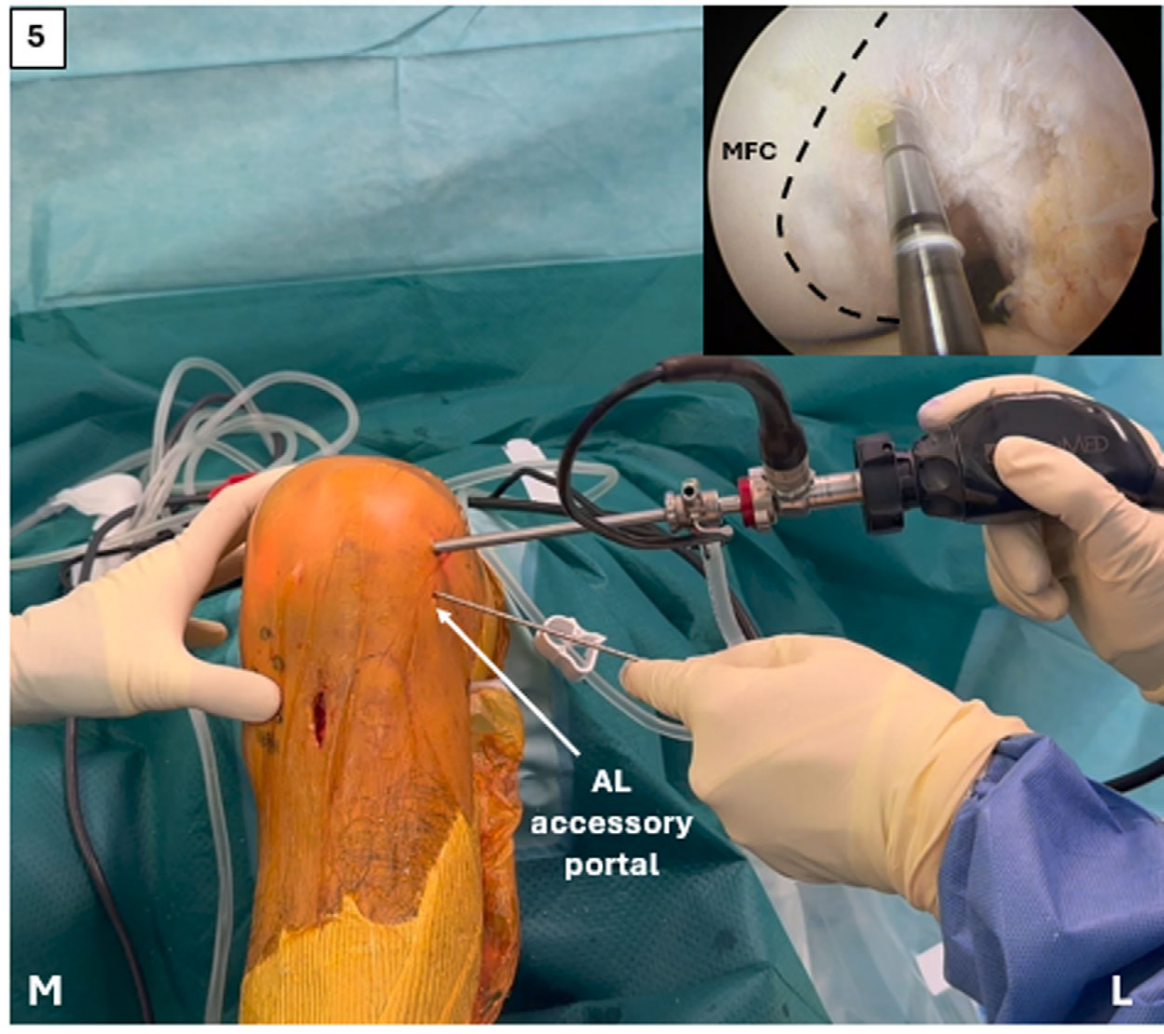
Intraoperative photograph of the left knee in 90° of flexion showing creation of the PCL femoral tunnel. Under direct arthroscopic visualization through the AL portal, a guidewire is introduced through an accessory anterolateral portal and inserted at the native PCL footprint, approximately 10 mm posterior to the articular cartilage of the medial femoral condyle. The guidewire is directed proximally and slightly posteriorly to the lateral femoral epicondyle then it is over‐drilled from inside to outside using a 9 mm drill‐bit to create a 20 mm length femoral socket. (AL, anterolateral; L, lateral side; M, medial side; MFC, medial femoral condyle; PCL, posterior cruciate ligament.)

The hook of the PCL tibial guide (Anatomic Contour PCL Guide, Left, Arthrex, Naples, FL, USA) is introduced through the AM portal toward the native PCL tibial footprint under direct arthroscopic visualization through the PM portal. A guidewire is then inserted from the ST insertion site to the PCL tibial footprint, and its intra‐articular position is checked under direct arthroscopic visualization once the guidewire tip becomes visible (Video 1). The guidewire is then over‐drilled subsequently with a 4, 5‐ and 9‐mm cannulated reamer to create the tibial tunnel (Figure 6). A bent 2.0 FiberStick suture (Arthrex, Naples, FL, USA) is then passed through the tibial tunnel and retrieved from the anterolateral portal.

**FIGURE 6:**
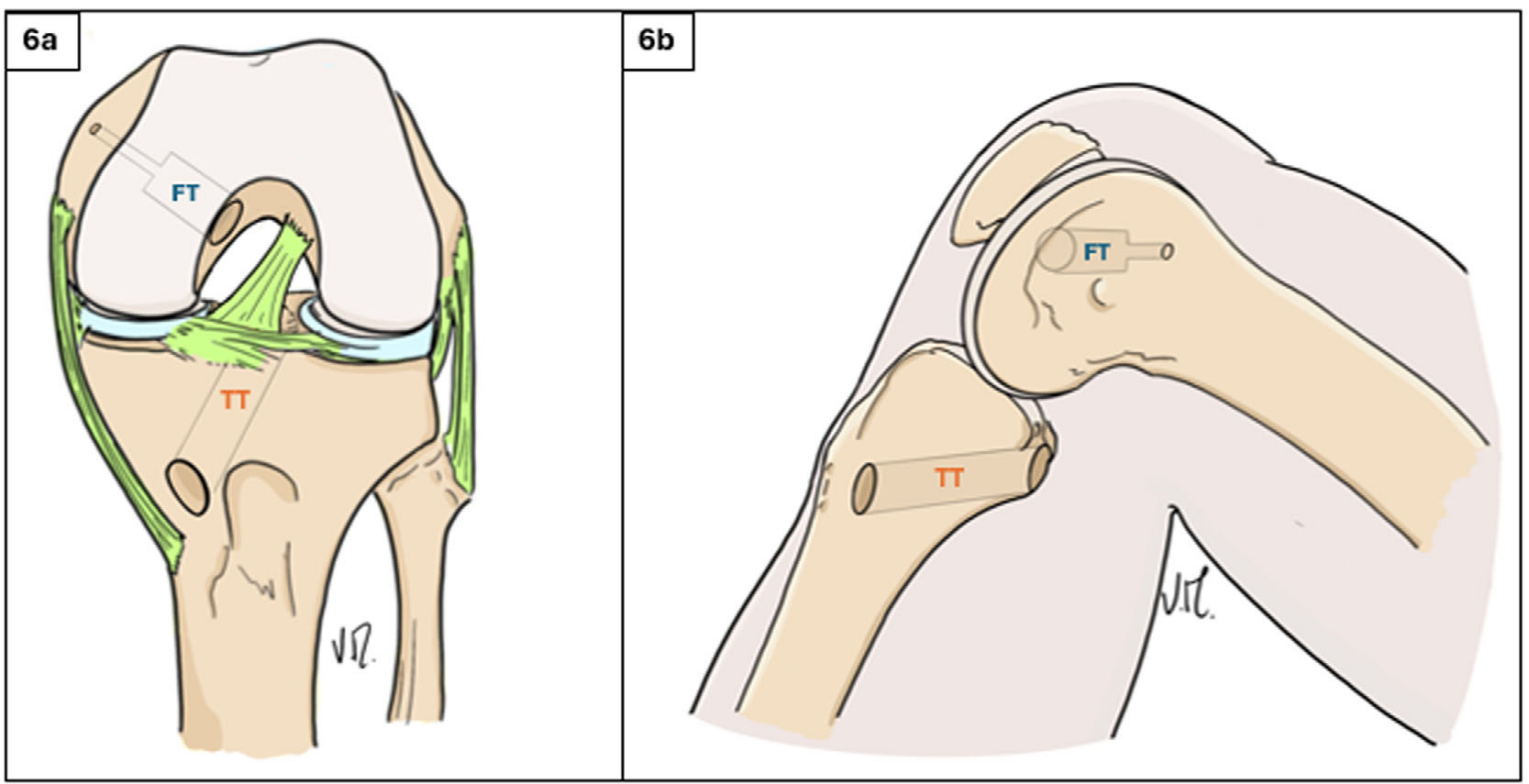
Schematic illustration of the left knee joint in anteroposterior (a) and lateral (b) views showing the created tibial and femoral tunnels for PCL graft reconstruction. (FT, femoral tunnel; PCL, posterior cruciate ligament; TT, tibial tunnel.)

### Pedicled PCL Graft Preparation

Accurate determination of the graft length is fundamental to obtain proper graft tension from the tibial insertion to the femoral tunnel apex. A green suture is passed through the tibial tunnel using the previously placed loop suture and advanced until reaching the base of the femoral socket under direct arthroscopic visualization via the AM portal. A clamp is then applied to the green suture at the level of the ST tendon insertion. The distance (D1) between the clamp and the intra‐articular part of the green suture within the femoral socket represents the required graft length from the ST insertion to the base of the femoral socket. In addition, a hook is used to measure the distance (D2) from the ST tendon insertion to the intra‐articular exit of the tibial tunnel (Figure 7a).

**FIGURE 7:**
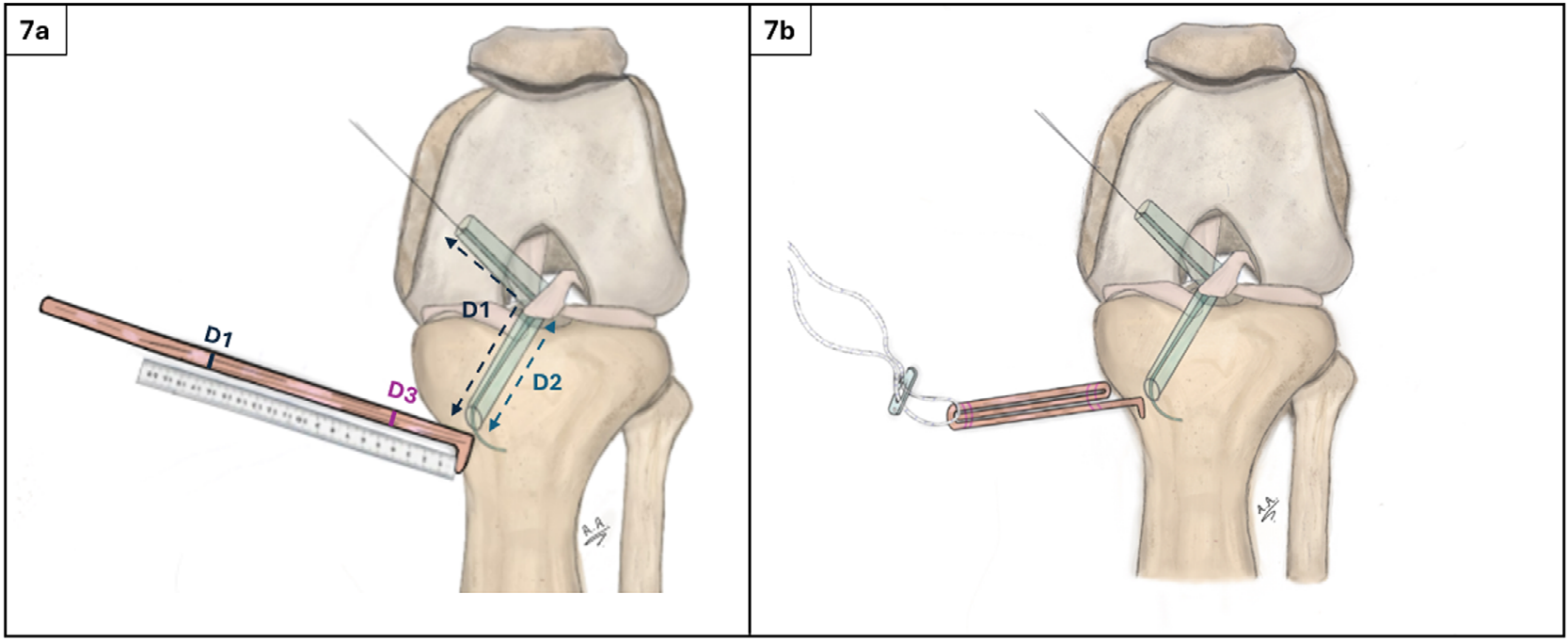
Schematic illustration of the left knee in 90° of flexion, showing the preparation of the pedicled PCL graft. A first mark is made on the ST tendon at a distance D3 = D2‐2 cm with the 2 cm representing the tripled part of the graft intended to be within the tibial tunnel. A second mark is made at D1 from the ST tendon insertion (a). D1 represents the required graft length from the ST insertion to the base of the femoral socket. D2 represents the distance from the ST tendon insertion to the intra‐articular exit of the tibial tunnel. A tripled ST tendon graft is folded and sutured to itself between both marks based on the thickness of the ST tendon and looped with an adjustable Tightrope RT implant (Arthrex, Naples, FL, USA) (b). (PCL, posterior cruciate ligament; ST, semitendinosus.)

A first mark is made on the ST tendon at a distance D3 = D2‐2 cm with the 2 cm representing the tripled part of the graft intended to be within the tibial tunnel. A second mark is made at D1 from the ST tendon insertion (Figure 7a).

A tripled ST tendon Graft is folded and sutured to itself between both marks based on the thickness of the ST Tendon and looped with an adjustable Tightrope RT implant (Arthrex, Naples, FL, USA) (Figure 7b).

### PCL Graft Passage and Fixation

The prepared tripled, pedicled ST PCL graft is passed through the tibial tunnel using the FiberLoop suture, with the TightRope RT device (Arthrex, Naples, FL, USA) pulled into the femoral tunnel under direct arthroscopic visualization via the AM portal and then positioned and securely fixed on the medial femoral condyle. The graft is gradually pulled to fill the femoral tunnel (Video 1).

With the knee at 90° flexion, posterior tibial translation is reduced, and the PCL graft is fixed with 2 interference screws (FastThread, Arthrex, Naples, FL, USA) to ensure accurate tibial fixation. The first 9 mm × 25 mm interference screw is inserted until the posterior cortex of the tibia, and the second 9 mm × 25 mm interference screw is inserted flush to the anterior entry of the tibial tunnel (Figure 8).

**FIGURE 8:**
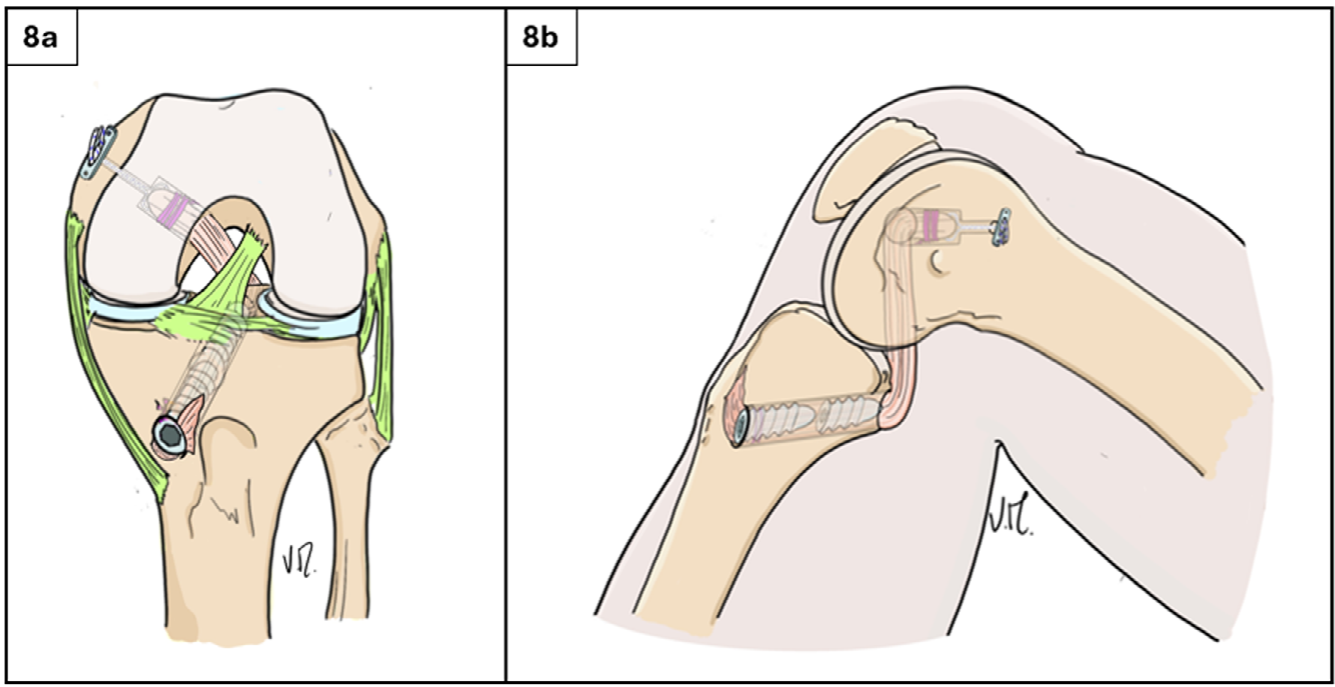
Schematic illustration of the knee joint in anteroposterior (a) and lateral (b) views showing the result after the completion of the pedicled tripled PCL reconstruction with triple tibial fixation. (PCL, posterior cruciate ligament.)

### Postoperative Protocol

The postoperative protocol consists of PCL knee brace immobilization for 6 weeks and partial weight bearing with crutches is allowed while keeping the knee locked in extension. Passive range of motion exercises are initiated early in the prone position to reduce posterior tibial loading. Full weight bearing is allowed after 6 weeks with crutches and an unlocked brace. The patient is allowed to return to sports, starting with nonpivoting activities at 6 months, followed by pivoting sports at 9 months postoperatively.

## DISCUSSION

PCL reconstruction remains a technically challenging procedure that requires advanced surgical skills to avoid intraoperative pitfalls. In this article, we describe a simplified technique for PCL reconstruction using a ST tendon autograft, which is maintained attached to its tibial insertion to preserve graft vascularity, and enhance biological healing.^8^ Additionally, using the trans‐septal approach provides an optimal visualization of the posterior compartments and precise preparation of the PCL tibial tunnel footprint. It also allows preservation of the PCL remnants and meniscofemoral ligament, which are crucial for enhancing graft healing, improving knee stability and preserving native mechanoreceptors involved in proprioceptive function.^11^, ^12^, ^13^ Unlike other described techniques,^14^, ^15^ this technique allows radiation‐free PCL reconstruction, as fluoroscopic guidance is not required for tibial tunnel creation. It is less time‐consuming, with easier patient positioning and draping and may reduce the risk of infection.

Triple tibial fixation of the PCL graft is achieved by preserving its native tibial attachment and by using 2 interference screws within the tibial tunnel. The aim of this construct is to prevent graft laxity and ensure optimal anteroposterior stability.

The limitations of our technique include the learning curve associated with the trans‐septal approach, which must be mastered to minimize neurovascular risks. Additionally, this technique is not feasible in revision procedures where the hamstring tendons are absent, or when the ST tendon is insufficient in diameter or length to create tripled graft. Precise measurement of the tibial and femoral tunnel lengths under direct arthroscopic visualization is essential to avoid graft‐tunnel length mismatch, which could compromise the final functional outcome.

In our perspective, we believe that our technique provides an accurate PCL reconstruction with preservation of the native remnants and the use of vascularized pedicled ST graft, allowing optimal graft integration and improved final knee stability.

## DISCLOSURES

The author (E.C.) declares the following financial interests/personal relationships which may be considered as potential competing interests: E.C. reports a relationship with Arthrex that includes consulting or advisory. The other authors (A.A., V.M., D.M., L.O., A.L.C., R.P.) declare that they have no known competing financial interests or personal relationships that could have appeared to influence the work reported in this paper.
